# Prediction of 3D Protein Structure Based on The Mutation of *AKAP3* and *PLOD3* Genes in The Case of Non-Obstructive Azoospermia

**DOI:** 10.22074/ijfs.2020.6028

**Published:** 2020-07-15

**Authors:** Ajit Kumar Saxena, Meenakshi Tiwari, Mukta Agarwal, Aprajita Aniket Kumar

**Affiliations:** 1 Department of Pathology/Laboratory Medicine, All India Institute of Medical Sciences, Bihar, India; 2 Department of Obstetrics and Gynaecology, All India Institute of Medical Sciences, Bihar, India

**Keywords:** *AKAP3*, Infertility, Iterative Threading ASSEmbly Refinement, *PLOD3* gene, Whole Exome Sequencing

## Abstract

**Background:**

The present study has been designed with the aim of evaluating A-kinase anchoring proteins 3 (AKAP3) and Procollagen-Lysine, 2-Oxoglutarate 5-Dioxygenase 3 (*PLOD3*) gene mutations and prediction of 3D protein structure for ligand binding activity in the cases of non-obstructive azoospermic male.

**Materials and Methods:**

Clinically diagnosed cases of non-obstructive azoospermia (n=111) with age matched controls (n=42) were included in the present case-control study for genetics analysis and confirmation of diagnosis. The sample size was calculated using Epi info software version 6 with 90 power and 95% confidence interval. Genomic DNA was isolated from blood (2.0 ml) and a selected case was used for whole exome sequencing (WES) using Illumina Hiseq for identification of the genes. Bioinformatic tools were used for decode the amino acid sequence from biological database (www.ncbi.nlm.nih.gov/protein). 3D protein structure of *AKAP3* and PLOD3 genes was predicted using I-TASSER server and binding energy was calculated by Ramachandran plot.

**Results:**

Present study revealed the mutation of *AKAP3* gene, showing frameshift mutation at rs67512580 (ACT → -CT) and loss of adenine in homozygous condition, where, leucine changed into serine. Similarly, PLOD3 gene shows missense mutation in heterozygous condition due to loss of guanine in the sequence AGG→A-G and it is responsible for the change in post-translational event of amino acid where arginine change into lysine. 3D structure shows 8 and 4 pockets binding site in AKAP3 and PLOD3 gene encoded proteins with MTX respectively, but only one site bound to the receptor with less binding energy representing efficient model of protein structure.

**Conclusion:**

These genetic variations are responsible for alteration of translational events of amino acid sequences, leading to protein synthesis change following alteration in the predicted 3D structure and functions during spermiogen- esis, which might be a causative “risk” factor for male infertility.

## Introduction

Globally, infertility is a serious problem in the world, which affects more than 15% of the couples amounting to 48.5 million people. The genetic landscape of male infertility is extremely heterogeneous due to molecular interactions that exist between primary spermatocytes, spermatids and finally sperm. More than 2000 genes work together in synchronous way to form single mature and healthy sperm under highly complex procedure -spermatogenesis. Amongst more than 15% of infertile populations, males alone contribute to 20-30% cases of infertility. It has been suggested by various studies that genetic mutations are responsible for dysregulation of spermatogenesis leading to male infertility (1-3). The highest frequency (25%) was observed in the nonobstructive azoospermic category. Simultaneously, the other anomalies were also evaluated on the basis of semen analysis in the cases of oligozoospermia (4). The cellular morphogenetic events undergo drastic changes including chromatin condensation, acrosome formation and maturation of sperm tail (5). Whole exome sequencing (WES) is one of the most sensitive and powerful technique to generate mutational spectra of unidentified gene(s) and their regulation in disease condition such as infertility. DNA sequencing analysis help identify nucleotide changes such as insertion, deletion or frameshift/non-frameshift mutation that alters post-translational event resulting in modifications in proteins structure and function. They might interfere in the process of spermatogenesis relevant role in male infertility. Identifying such changes might help determine the causative factors involved in unexplained infertility. Our candidate genes namely A-kinase anchoring proteins 3 (*AKAP3*) and Procollagen-Lysine, 2-Oxoglutarate 5-Dioxygenase 3 (*PLOD3*) were collected from the affected “gene pool” of infertile cases after whole genome sequencing and predicted 3D model protein structure for ligand binding receptor site.

In human, investigations showed several isoforms of AKAP gene family express in testicular tissue, among them sperm-specific AKAP3 were found to localize in the sperm tail and regulate sperm motility. The main functions of *AKAPs* are to modulate protein kinase A (PKA) signalling, during germ cell proliferation and further development of the gamete. *In vitro* studies suggested that *AKAP3* interacts with other isoforms of AKAPs and plays an important role during assembly of fibrous sheath and spermatid morphogenesis (6). However, the roles of *AKAP3* gene expression to modulate PKA functions are still confusing due to the lack of define structure during spermiogenesis. *In vitro* studies on protein interaction indicated that *AKAP3* gene has been associated with numerous signalling proteins, like PDE4A, Ga13 and Ropporin, which participate in the regulation of sperm motility (7, 8).

Similarly, *PLOD3* gene plays an important role in spermatogenesis and mutation in this gene has been associated with connective tissue disorder and congenital malformations (6). PLOD3 gene encodes Lysyl hydroxylase 3 (LH3), as an enzyme with multiple functions that leads to hydroxylation of lysyl residues and O-glycosylation of hydroxylysyl. Such reactions leads to production of monosaccharide or disaccharide derivatives that play role in post translational modifications involved in collagen biosynthesis. Previous studies have suggested the significance of *PLOD3* gene in biosynthesis of glycosylated type IV and VI collagens required for normal formation of basement membranes, however, the functional role of this enzyme is not clear yet (7). In testis, Sertoli cells and germ cells are in close contact with the basement membrane which is a modified form of extracellular matrix (ECM). These cells are relaxed on the basement membrane of the seminiferous tubule for hormonal supports at different stages of the seminiferous epithelial cycle. However, the role of ECM is poorly understood in regulating spermatogenesis (9).

There is lack of information in the literatures, regarding the *AKAP3* and *PLOD3* genes, their structural and functional interaction on the proliferating germ cells during translational events and how to play a significant role in reproductive dysfunction. However, WES data analysis confirms the mutation types in male infertility. Therefore, this study is quite important to understand the molecular pathogenesis of pre- and post-translational events during spermiogenesis, in addition to help predict 3D model structure of protein after bioinformatics tools like molecular docking to methotrexate with respect to controls. Hence, the present study explores knowledge of functional genomics in reproductive medicine.

## Materials and Methods

The present study has been performed in clinically diagnosed patients (n=111) of non-obstructive azoospermia (NOA) classified after semen analysis, according to WHO guidelines (2010) with respect to the age matched controls (n=42) (3). Inclusion criteria for present study is that none of them had any history of childhood disease, radiation exposure or prescription of continuation of drug and the median age of patients was 35.4 years old (age group range 21-45 years). The sample size was calculated using Epi info software version 6 with 90 power and 95% confidence interval (CI), with alpha error 0.05%; beta 0.02 taking into account the normal population have prevalence of gene mutation in male infertile cases that varies from 1.0 to 3.5%. This study was further extended in the case of NOA to identify further “novel” mutations. The genetics analyses were carried out in the Department of Pathology/Lab Medicine, All India Institute of Medical Sciences, Patna. Blood samples (2.0 ml) were collected from the proband after written informed consent, and the study was approved by the Institutional Ethical Committee (IEC) (code: dean/2008-09/384). The bioinformatics tool were used for the prediction of 3D protein helical structure and their functional binding site to the ligand (drug) with calculated energy using docking (server) system.

### Identification of *AKAP3* and *PLOD3* genes from whole exome sequencing

Genomic DNA was isolated from the clinically diagnosed cases of male infertility for characterization of Y-chromosome microdeletion using STS markers (3). A selected case was used for further characterization of small insertions/deletions (In/Dels) and single nucleotide variants (SNVs) using WES by Illumina Hiseq 2000 (Illumina, USA). These variants were further characterized using filters covering position of the gene variants excluded non-coding and repetitive regions (10). The information of *AKAP3* and *PLOD3* genes and translational events were further verified on the basis of availability of sequence database (https://www.ncbi.nlm.nih.gov/protein). Hence, prediction of 3D structure of *AKAP3* gene becomes quite relevant as it is not available in the structural database (https://www.rcsb.org/). Similarly, *PLOD3* protein 3D structure was further predicted on the basis of sequencing data for remodelling of chromatin during spermiogenesis into ligand binding sites to explore the pathogenesis of infertility.

### Homology modelling of 3D structure

I-TASSER (Iterative Threading ASSEmbly Refinement) is used to evaluate the structure and function of protein, after prediction in scientific research based on state of theart of algorithms (11). First structural template is identified by using local meta threading server (LOMETS) from the construction of full-length atomic models and iterative template-based fragment assembly from protein data bank (PDB). I-TASSER have five models for prediction of large clusters of protein structure. For prediction of protein structure, in each model C-score is calculated (1.19) on the basis of significance of alignment and convergence parameters. Higher value of C-score range (5 to 2) signify the best structure of protein. Structural similarity is calculated by template modelling score (TM; 0.57 ± 0.15) between query and template protein using root mean square deviation (RMSD; 11.4 ± 4.5Å0) between amino acid residues and protein length following the correlation observed between these qualities to improve the predicted 3D model (12,13).

### Identification of the binding site of 3D model structure

The ligand binding sites are active site of enzyme during assembly of protein structure and become relevant to explore the functional interaction to other molecule. During structural analysis, the strategy was initiated with identification of the target molecule to ligand binding sites (pocket) including donors and acceptors of potential hydrogen bond that are hydrophobic in nature. In protein structure, there are well accepted target bind sites to ligand which are highly specific in different disease conditions. Prediction of 3D protein structure is developed from the sensitive template library (http://raptorx.uchicago.edu/ bindingsite/) and arranged the target sequence based on neural networking (https://playmolecule.org/ deepsite/) (14-16).

### Selection of methotrexate as ligand binding molecule

Activity of methotrexate (MTX), as an antagonist of folate that inhibits tetrahydrofolate dehydrogenase enzyme, is essential for DNA synthesis (https://www. drugbank.ca/drugs/DB00563). Selection of MTX has been developed not only due to the commonly used as an antineoplastic agent for the management of malignancy, but also used as an immunosuppressive drug. MTX is highly toxic in nature and entered into the S-phase of cell-cycle affecting rapidly dividing cells, which leads to inhibit DNA replication followed by cell-death (17). The present study becomes relevant as it proposes an approach to reduce the cellular toxicity by structural remodelling of ligand binding sites during gene-protein or drug-protein interaction in rapid dividing cells e.g. germ cells.

### Molecular docking of *AKAP3* and *PLOD3* protein

The iGEMDOCK v2.1 software (BioXGEM Lab, Taiwan) was used for evaluation of protein structural and functional activities, based on algorithm and scoring efficiency between standalone. iGEMDOCK v2.1 is an integrated software used for structural analysis and pharmacological interaction with the corresponding ligand molecules. Findings of the software showed interaction of the biological active compounds involved in biological mechanisms. This software has embedded statistical application for calculation of minimum energy to binding sites. It automatically generates pharmacological interaction and calculates the preference between hydrogen atoms and ligand binding site with the help of compound library. Furthermore, RasMol (visualization tool for protein-ligand interactions) displays interactions with conserved residues of amino acid and the functional groups of compound. Thus, iGEMDOCK provides an interactive boundary for visualizing the active compound by combining the pharmacological interactions in the energybased scoring function (18). The visualization properties (analyses) inside the helical structure of protein to the ligand binding sites (enlarge view) are concluded by using UCSF Chimera technique (19).

### Statistical Analysis

Chi square (x^2^) test (two-tailed) was applied to find out significant differences (P values) between the infertile cases and controls.

## Results

**Fig 1:**
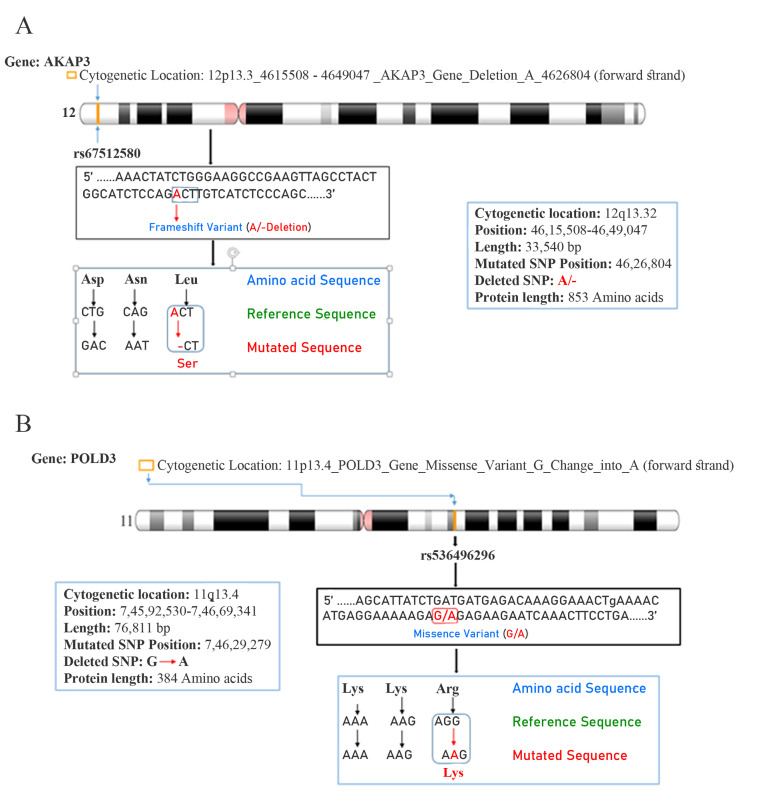
Gene mapping-chromosomal location. Cytogenetic location and mutational site of the **A.** *AKAP3* and **B.** *PLOD3* genes mapped on chromosomes 12p13.32 and 11q13.4, respectively (https://www.ncbi.nlm.nih.gov/genome/tools/gdp).

In our recent study, we identified that deletion frequency of AZFa region is 1.0%, while AZFb and AZFc regions respectively showed 6% and 19% in non-obstetric azoospermic cases. We further extended our study to identify novel gene mutations followed by bioinformatics analysis in the cases of NOA. In the present study our candidate genes, *AKAP3* and *PLOD3* were selected after sequencing analysis and these mutations further characterized translational event after using bioinformatics tools in the case of infertility. Figures 1 A and B show location of *AKAP3* gene mapped on chromosome 12p13.3 (variant table NC_000012.12) with the loss of adenine resulting in modifications of translation process of amino acid (i.e. leucine change into serine) due to “frameshift mutation” at rs67512580 (ACT →-CT) in homozygous condition. Similarly, PLOD3 gene locus is on chromosome 11p13.4 (variant table GCF_000001405.39) showing “missense mutation” at rs536496296 in heterozygous condition, where the nucleotide ‘G’ is missed in sequence AGG→A-G. This results in changes in translational event of spermiogenesis (i.e. amino acid arginine is changed into lysine). *PLOD3* gene mapped on chromosome 11, showing “missense mutation”, where, guanine is changed into adenine (G → A), followed by changes in the amino acid arginine into lysine.

**Fig 2:**
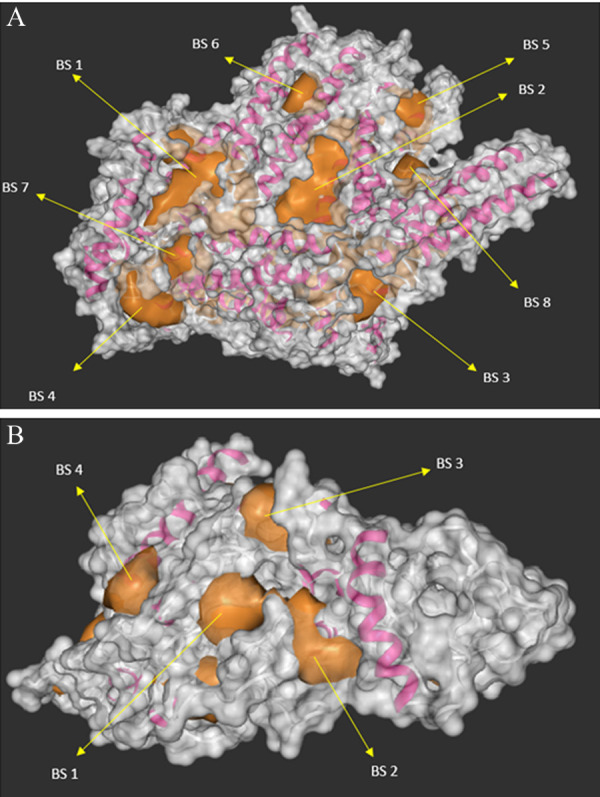
3-D protein structure of *AKAP3* and PLOD3 genes. Illustration of the modelled 3D structure and available target protein of different binding sites with α-chain and β-sheets (magenta), binding pocket (golden) and surface structure visualization (grey) of **A.** *AKAP3* and **B.** *PLOD3* protein structure binding sites are represented by arrow (→).

**Fig 3:**
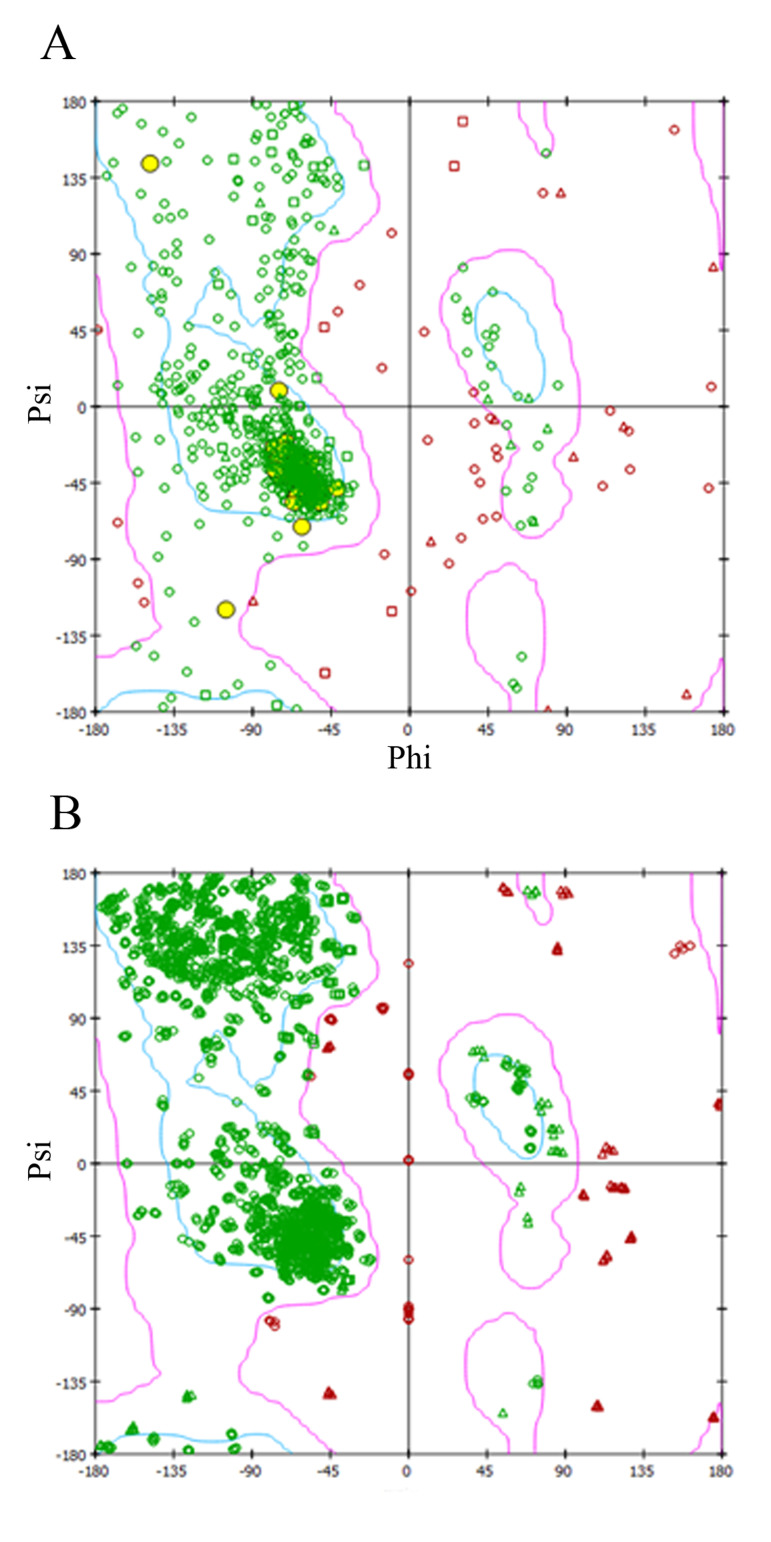
Ramachandran plot. Homology modelled structure between **A.** *AKAP3* and **B.** *PLOD3* gene coded proteins, supported by Ramachandran plot.

**Fig 4:**
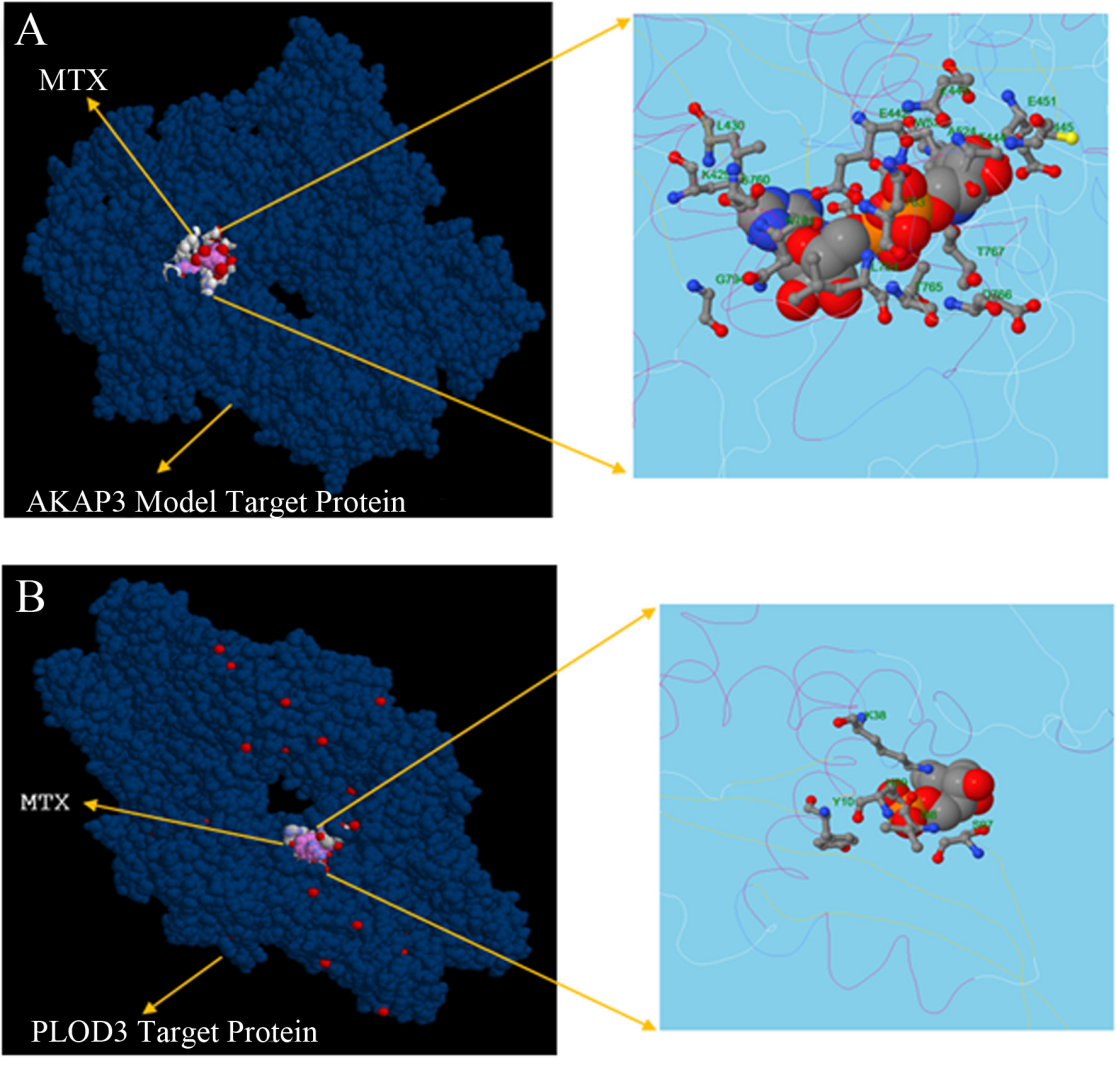
MTX binding with AKAP3 and PLOD3 protein structures. Structure showing the binding site with MTX molecule superimposed on target protein. Structure models of **A.** *AKAP3* and **B.** PLOD3 are showed in enlarged view as visualized by UCSF chimera.

Due to the lack of normal structure for AKAP3 protein in the structural data bank, iTASSER server was used for protein homology modelling using 6BFIA.pdb as a template. Similarly, PLOD3 normal protein structure prediction was based on 3E0J.pdb template as shown in supplementary Figures 1A and B. The predicted 3D modelled of targeted protein showed different binding sites to α-chain and β-sheets (magenta). This also reveals eight and four pocket binding pocket (golden) as predicted in Figures 2 A and B for both of the AKAP3 and PLOD3 proteins, respectively. Table 1 shows molecular docking between normal and mutated protein with MTX as shown to the active binding sites with their residues through VDW and H-bonding, with one active site (represented in bold letter) which gives significant binding site to the receptor and inhibits unbalanced functions of the protein molecule. The gene coded 3D modelled protein structure and their binding energy with favoured regions lies in phi (Φ) and psi (Ψ), which further confirms active stability of residue after construction of Ramachandran plot, as shown in Figures 3.A and B for AKAP3 and PLOD3 proteins, respectively. Molecular docking was done using iGEMDOCK to study the protein drug interaction. Interestingly, free binding energy in the mutated protein structure of AKAP3 and PLOD3 with MTX was between 0 to -10 Kcal/mol, showing significant binding energy whereas the normal protein structures of AKAP3 and PLOD3 showed low free binding energy of -85.33 Kcal/mol and -132.5 Kcal/ mol respectively. This indicates weak binding efficiency between normal protein structures and MTX, as depicted in Table 1. Furthermore, findings of iGEMDOCK revealed less binding energy (10.03 and-8.40), VDW (Van der Waals forces) (21.89 and-23.07), H-bond (12.82 and-23.07), and Z-Score (4.07 and 3.10) were required to bind both mutated protein and ligand molecule. Although, less binding energy represent good potential to develop 3D structure based on drug designing and reduced mutagenic properties of AKAP3 and PLOD3 gene coded protein. Figures 4.A and B showed binding sites of MTX into the target protein and interaction with altered amino acid residues.

**Table 1:** Comparison of sperm parameters (± SD) between the experimental groups after frozen-thawed and treatment with 10 μg/ml Calligonum (CGM) extract and LIPUS (pulsed mode and continues wave)


DockingCompound	Binding Sites (BS)	Binding Site(Residues & Position)	Residues BindingEnergy (kj/mol-1)	-19.63vdm	H-bond	Z-Score

AKAP3 Normal Protein Structure with MTX Binding	BS	M286 T287	-85.33^*^	-65.61	-19.72	1.645
		A289 K324				
		Y288 D290				
AKAP3 Mutated Protein Structure with MTX Binding	BS 1	K429 L430	-10.03^*^	-21.89	-12.82	4.07
		E442 E443				
		T444 C445				
		E451 D521				
		S522 W523				
		A524 S760				
		N761 N763				
		L764 T765				
		D766 T767				
		G794				
	BS 2	D697 D698	+8.13	-18.22	-6.82	6.09
		S704 R705				
		D698 D702				
		A703 S704				
		P792				
	BS 3	E123 S150	+12.35	-10.48	-16.70	3.15
		H342 S343				
		T345				
	BS 4	S343 M349	+17.65	-17.33	-14.81	8.19
		T350				
	BS 5	E671	+28.12	-30.75	-11.07	^2.27^
	BS 6	T444 C445	+81.35	-09.34	-9.56	7.48
		A446				
	BS 7	Y435 E614	+72.58	-32.59	-19.32	5.67
		P615 K616				
	BS 8	F246 N250	+48.42	-45.63	-36.63	9.43
		S280 V281				
		I285 L378				
		Y382				
PLOD3 Normal Protein Structure with MTX Binding	BS	T50 T305	-132.5^*^	-92.8	-39.76	1.650
		P307 P379				
		D380 T381				
		T390 D391				
		F393				
PLOD3 Mutated Protein Structure with MTX Binding	BS 1	K38 S97	-8.40^*^	-23.07	-18.15	3.10
		I98 H99				
		Y101				
	BS 2	V34 N35	-01.46	-11.25	-15.09	2.15
		K38 Q39				
		Y42				
	BS 3	V77	+20.38	-17.49	-12.27	4.01
	BS 4	V34 N35	+67.42	-19.63	-15.32	1.15
		A134				


*; Standard molecular docking binding energy ranges from 0.0 to - 10.0 in mutated protein showing significant interaction with drug (MTX). MTX; Methotrexate , and VDW; Van der waals forces.

## Discussion

The present study explores acquaintance between changes of nucleotides (frameshift and non-frameshift mutation) in *AKAP3* and *PLOD3* gene and their transcriptional events (amino acids) in the cases of infertility. Human spermatogenesis is highly sensitive process that involves complex interactions between genetic and environmental factors. Such pathways regulate proliferation and differentiation of germ cells (spermatocytes, spermatids, sperm) and Sertoli cells inside the seminiferous tubules of testes. WES is one of the most powerful and sensitive techniques utilized for identification of new mutations in genome. Bioinformatics tools play a significant role in structural designing and modelling of drugprotein interactions based on pharmacogenomics and personalized medicine. Earlier study, based on WES and bioinformatics analysis gives new insight into discovery of gene(s) and disease(s). Additionally, role of single nucleotide polymorphism (SNP) mutations, such as In/Del, were identified using the ensemble database (https://www.ensembl.org/Multi/Search/ Results q=snp;site=ensembl_all) during germ cell differentiation and proliferation in spermatogenesis (10).

With the help of bioinformatics tools, we are able to trace our finding in various biological databases across the countries. However, there is lack of AKAP3 protein 3D structure availability in structural database (Protein Data Bank) (https://www.rcsb.org). Here, we used virtual protein modelling iTASSER server based on the principle of X-ray crystallography and Nuclear Magnetic Resonance. Hence modelling 3D structure of the mutated *AKAP3* gene coded protein with the help iTASSER online server and predicted the model. There are several available computational procedures for determination of protein structure in homologous modelling, but using this model we could perform the most accurate structural and functional predictions based on the algorithms (11). It firstly identified structural templates from the PDB and compared template-target based modelling (TTBM) with powered detection and alignment accuracy. With no doubt, another TTBM has become an extremely useful approach for the prediction of protein complex structure based on BLAST alignment methods. Prediction of 3D protein structure based on *AKAP3* and *PLOD3* gene sequences provide knowledge of structural and functional activities of the encoded protein compared to the normal protein. Ligand binding with specific active sites has been predicted with MTX, which is known to interfere with spermatogenesis. *AKAP3* gene plays significant role in sperm motility after stimulating effect of bicarbonate, which activates soluble adenylyl cyclase followed by triggering signalling cascade of tyrosine phosphorylation. *AKAP3* activates PI3K/Akt pathway, and leucine influences sperm motility. But in the present study, leucine change into serine might be one of the causative factors for infertility by interfering motility of sperm during process of fertilization (21). Another relevant *PLOD3* gene associated to infertility is a member of family of lysyl hydroxylase that catalyses hydroxylation of proline and lysine at the time of collagen synthesis. Collagen, as a major component of the ECM, plays an essential role in embryo implantation (22). Similarly, *PLOD3* gene mutation might interfere with translational event due to the change of arginine into lysine and failing catalysis of hydroxylation event during collagen synthesis, resulting in alteration of sperm function. Previously it was reported that oral supplement of arginine enhanced sperm count and motility in the majority of oligospermia cases and prevented infertility (23).

Interestingly, the bioinformatics tools using molecular docking studies help explore the structural (integrity) and functional prediction of protein structure to ligand binding sites affecting sperm morphology during spermiogenesis. However, it is not clear how the gene(s) interact with protein and protein interact with the ligand (drug), like methotrexate which known to function during germ cell proliferation and modify defined 3D structure followed by loss of function. Drugs bind to protein and specific binding target sites where they are firstly absorbed, secondly transported and finally distributed to their respective sites, if not mutated. Using iGEMDOCK v2.1, ligand binding site was predicted and based on the minimum energy to bind with MTX, the best model was chosen as shown in Table 1. Thus, present findings justify relevance of normal and mutated protein interactions with MTX during prediction of 3D model structure (*AKAP3* gene) developed and reported for the first time in the field of reproductive medicine (Fig. S1 A and B) (See Supplementary Online Information at www.ijfs.ir). However, our efforts are evolved to predict 3D structure and their affinities based on the binding energy to ligand after penetrance of mutated gene into the genome. This increases genetic susceptibility risk of the disease either in homozygous or heterozygous condition. WES is a highly sensitive and most reliable technique to identify new gene mutations in clinical samples. However, further validations are required to incorporate in large sample size, in order to make the study more significant.

## Conclusion

The findings of present study are quite interesting, as predicted structural and functional activities based on genomic alterations and germ cells proliferation during spermiogenesis. Such type of study widens the scope of developing new derivatives based on pharmacogenomics and personalized medicine for the management of infertility.
